# The impact of artificial intelligence perception on university students' academic engagement: the mediating role of academic motivation and the moderating role of academic self-efficacy

**DOI:** 10.3389/fpsyg.2026.1735410

**Published:** 2026-02-13

**Authors:** Yantao Shi, Haiyan Cui, Yuanchang Zhang, Xueli Hui, Guanghai Li, Mingkun Ouyang, Lingle Pan

**Affiliations:** 1 Faculty of Education, Guangxi Normal University, Guilin, China; 2 School of Education Science, Guangxi Minzu University, Nanning, China; 3 College of Biology and Food Engineering, Chuzhou University, Chuzhou, Anhui, China; 4 College of Preschool Education, Chongqing Youth Vocational & Technical College, Chongqing, China; 5 Zhejiang College of Security Technology, Wenzhou, China

**Keywords:** academic engagement, academic motivation, academic self-efficacy, generative artificial intelligence perception, university students

## Abstract

**Objective:**

To test whether academic motivation mediates the association between artificial intelligence (AI) perception and university students' academic engagement, and whether academic self-efficacy moderates the direct and indirect associations.

**Design:**

Cross-sectional, single-wave, self-administered questionnaire survey; all variables were assessed once at a single time point in a convenience sample of university students in Anhui, China.

**Method:**

A convenience sample of 1,484 university students in Anhui Province completed an anonymous questionnaire measuring AI perception, academic engagement, academic motivation, and academic self-efficacy. Descriptive statistics, correlations, and regression-based mediation and moderated mediation analyses were conducted.

**Results:**

AI-related perceptions were negatively associated with academic engagement, and academic motivation partially mediated this association. Academic self-efficacy moderated both the direct association and the indirect pathway via motivation: when self-efficacy was lower, the negative association between AI-related perceptions and engagement was stronger and the motivation–engagement association was weaker; when self-efficacy was higher, the opposite pattern emerged, and the indirect effect via motivation was stronger.

**Conclusion:**

AI-related perceptions were inversely associated with academic engagement partly through academic motivation, and these associations varied by academic self-efficacy. Given the cross-sectional, self-report design, the findings are correlational and require longitudinal or experimental verification.

## 1 Introduction

Academic engagement refers to a positive, fulfilling, and multidimensional psychological and behavioral state that students actively cultivate and sustain in their academic activities and extracurricular activities (Schaufeli et al., 2002; Maroco et al., 2016). Marôco et al. (2020) further divided it into three interrelated dimensions: cognitive input, emotional input, and behavioral input. According to Schaufeli et al. (2002), academic engagement is characterized by the core components of vigor, dedication, and absorption. It manifests in the academic process as the sustained mobilization of learners' cognitive, emotional, and behavioral resources, coupled with a enduring commitment to academic goals. Previous research has shown that students' academic engagement and academic experiences are closely related to academic motivation. Individuals with high academic engagem5nt exhibit higher academic experiences and motivation (Xie et al., 2020; Martin, 2009), and demonstrate a greater propensity to consistently regulate cognitive, emotional, and behavioral resources in academic tasks (Fredricks et al., 2004). Additionally, students' academic engagement also affects their academic attitudes and academic performance (Çali et al., 2024). Compared to students with high academic engagement, those with low engagement are more reliant on technological tools, which results in a lack of academic motivation during the academic process (Sampasa-Kanyinga et al., 2022). Furthermore, both internal and external factors are closely related to academic engagement. Individual characteristics (e.g., intrinsic motivation and self-efficacy) have been correlated with academic engagement (Bai et al., 2022; Pintrich and De Groot, 1990). External factors, particularly students' perceptions and acceptance of emerging technologies, may emerge as critical determinants of academic engagement (Skinner et al., 2008).

Artificial Intelligence (AI) is a technology based on complex deep academic architectures that can generate content simulating human creativity (including text, images, videos, etc.) in response to diverse instructions, such as voice commands, operational guidelines, or query questions (Tang et al., 2025a,b). Since 2022, numerous generative AI tools and platforms have been developed globally. AI has swept across the globe, particularly making a significant impact in the field of education (Chiu, 2023; Cooper, 2023; Jovanović and Campbell, 2022; Tang et al., 2025a,b). The “AI Perception Intensity” defined in this study does not refer to behavioral-level technology dependence or over-reliance. It refers to an individual's attitudinal evaluation and belief representation of AI, and the intensity of subjective perception that it may have an impact on their academic. Therefore, results obtained based on the USAP scale reflect an individual's cognitive representation level of the “influence–value” of AI, and should not be directly extrapolated as behavioral outcomes such as “excessive use,” “cognitive laziness,” or “superficial academic.” Relevant studies indicate that the psychological salience of AI in an individual's life, academic, and work largely determines their subjective perception and evaluation of this technology (Chan and Hu, 2023). Novelty, complexity, and transformative impact constitute the three factors for measuring AI perception intensity (Amofa et al., 2025; Lee et al., 2025; Capraro et al., 2024). The greater the novelty, complexity, and trans-formative impact, the higher the perceived intensity of AI, and the more significant its influence on people's cognition, behavior, and decision-making (Bai et al., 2025; Reding and Wells, 2022).

The perception of artificial intelligence (AI) has a negative impact on university students' academic engagement. According to the Technology Dependency Theory, when individuals perceive AI as having a high level of influence, their instrumental tendency to use it may increase (Davis, 1989). While positive perceptions may reduce perceived effort costs, under conditions of high pressure or insufficient self-regulation, heightened perception intensity may lead students to rely on AI to complete tasks, thereby reducing deep cognitive processing and diminishing academic engagement. Relevant studies have shown that when learners frequently use AI to generate answers or complete academic tasks, it may reduce the opportunities for active construction and deep processing of knowledge, which is associated with a higher level of academic burnout and task-avoidance tendencies (Zhai et al., 2024; Moorhouse and Kohnke, 2024; Gerlich, 2025; Zhang et al., 2024; Xu E. et al., 2023). Furthermore, in complex problem-solving contexts, individuals tend to choose the path of least cognitive load rather than engaging in active thinking and reflection, demonstrating a degree of “cognitive laziness” (Criado-Perez et al., 2024; Kool et al., 2010). Students who rely excessively and uncritically on AI-generated outputs may face a higher risk of academic integrity concerns (e.g., unacknowledged use of AI-generated text), regardless of their level of AI awareness (Yan, 2023; Zhang et al., 2025). In view of this, this study proposes that when students have a high evaluation of the “influence” of AI, the direction and level of their academic engagement may show significant situational dependence. In the absence of effective academic strategies and self-regulation support, academic engagement is more likely to be adversely affected (Backfisch et al., 2021; Xu et al., 2024).

Although existing research has made preliminary explorations into the impact of artificial intelligence (AI) perception on university students' academic engagement and drawn some conclusions, these studies often remain superficial and fail to deeply analyze the psychological and behavioral mechanisms (such as mediating and moderating effects) through which AI perception influences students' academic engagement. To address this research gap, this study plans to construct a moderated mediation model, aiming to explore the mediating role of academic motivation between excessive reliance on AI and students' academic engagement, as well as how academic self-efficacy moderates this relationship. This model holds theoretical value and seeks to answer the following questions: How does the perception of AI influence university students' academic engagement? Under what conditions does this influence become more significant?

### 1.1 Mediating role of academic motivation

Academic motivation refers to learners' acquired cognitions regarding competence beliefs and task value, their orientation toward success, and the positive or negative affective experiences elicited by self-evaluation (Fallah et al., 2020; Yaghoobi et al., 2025). Drawing on Self-Determination Theory (SDT) and the Technology Acceptance Model (TAM), academic motivation is not only associated with the extent to which basic psychological needs—autonomy, competence, and relatedness—are satisfied, but may also covary with learners' perceptual attitudes toward technological tools, namely their subjective appraisals of whether generative artificial intelligence (AI) is “more use yields more benefit,” how easy it is to use, and how salient its impact is in academic contexts (Deci and Ryan, 1985; Davis, 1989). Social cognitive theory, expectancy–value theory, control–value theory, and accounts of self-regulated academic and cognitive offloading converge on a similar negative explanation: when core cognitive processes are excessively outsourced to AI, students' sense of agency and efficacy experiences, expectations of success and task value, perceived control over academic, and investments in self-regulatory effort may be undermined, thereby compromising the maintenance of academic motivation. We propose that university students' perceptual attitudes toward AI may be negatively associated with their level of academic motivation. When students more strongly perceive AI as enabling efficient task completion, they may become more inclined to delegate key academic components—such as information searching, inferential processing, and text generation—to the tool, thereby reducing opportunities for autonomous exploration and independent problem solving. As a result, learners may experience a weaker sense of autonomy and control over the academic process, psychological experiences that are closely tied to intrinsic motivation (Bandura, 1997; Liu et al., 2023). Consistent with this account, prior studies indicates that a stronger tendency toward dependence on technological tools may co-occur with lower academic motivation (Abbas et al., 2024; Kuş, 2025). This viewpoint is supported by prior research indicatesing that excessive reliance on technological tools may erode individuals' intrinsic motivation and critical thinking capacity (Backfisch et al., 2021; Huang et al., 2020; Pajares and Miller, 1994). Greater use of technological tools may be accompanied by a preference for more surface-level cognitive processing and reduced investment in problem solving, which is in turn associated with lower levels of academic motivation (Gerlich, 2025; Jose et al., 2025).

Academic motivation may be positively associated with university students' academic engagement. The job demands–resources (JD–R) model indicates that positive qualities (such as academic motivation, self-efficacy, optimism, and empathy) can be conceptualized as key personal resources, which are associated with higher academic engagement and facilitate more effective coping with academic demands and improved academic effectiveness (Bakker et al., 2004, 2007; Xanthopoulou et al., 2007). A recent meta-analysis further indicates that academic motivation is significantly and positively related to academic engagement (Vu et al., 2024). Academic motivation is commonly defined as the driving force underlying students' participation in academic activities and is linked to higher levels of interest and effort, as well as greater vigor, absorption, and dedication (Singh et al., 2022; Liu et al., 2024; Chen et al., 2023a,b; Singh et al., 2022). Students with higher levels of academic motivation are typically more willing to proactively participate in academic activities and tend to invest more time and effort, exhibiting higher levels of academic engagement (Li and Xue, 2023; Singh et al., 2002). Moreover, prior research has shown that stronger academic motivation is associated with more active thinking and deeper processing of academic problems (cognitive dimension; Xu Z. et al., 2023), more frequent experiences of positive academic-related emotions (affective dimension; Liu et al., 2024; Valiente et al., 2012), and more proactive participation in classroom interactions (behavioral dimension; Fredricks et al., 2004).

Based on the foregoing theories and prior empirical evidence, we propose the following hypotheses:

**H1:** University students' perceptual attitudes toward generative artificial intelligence (AI) will be significantly and negatively associated with their academic motivation.

**H2:** Academic motivation will be significantly and positively associated with university students' academic engagement.

**H3:** Academic motivation will mediate the association between perceived AI attitudes and university students' academic engagement.

### 1.2 Academic self-efficacy as a moderator: the conditional association between perceptions of AI and academic engagement

Academic self-efficacy refers to individuals' beliefs in their capability to learn at a specified level and successfully accomplish academic tasks (Bandura, 1997; Schunk and DiBenedetto, 2020; Yi et al., 2025). Prior research indicates that self-efficacy is commonly regarded as a key noncognitive factor associated with academic motivation, academic processes, and educational outcomes (Pajares, 1996a,b), and a substantial body of evidence consistently shows a positive relationship between self-efficacy and academic performance (Multon et al., 1991; Honicke and Broadbent, 2016). However, research examining the potential indirect effects of academic self-efficacy on academic behavior (e.g., moderating effects) remains comparatively limited (Wang et al., 2024; Zhou, 2022). Accordingly, the present study hypothesizes that academic self-efficacy may moderate both the direct association between perceived AI and academic engagement and the potential indirect pathway linking perceived AI to engagement.

First, we propose that academic self-efficacy may moderate the association between academic motivation and university students' academic engagement. Social cognitive theory indicates that academic behavior emerges from the dynamic interplay between personal factors and situational factors (Aquino et al., 2009; Bandura, 2001). Prior research has shown that academic self-efficacy, as a key personal factor, is positively associated with academic engagement among university students (Meng and Zhang, 2023). However, evidence remains limited regarding whether and how the interaction between academic self-efficacy and academic motivation is linked to variability in academic engagement. Drawing on social cognitive theory, we therefore infer that, compared with students low in academic self-efficacy, those with higher academic self-efficacy may exhibit a stronger positive association between academic motivation and engagement. Higher academic self-efficacy implies stronger beliefs in one's capability to accomplish academic tasks and greater expectations of success (Bandura, 1997), which are likely to translate into greater willingness to participate in tasks and increased investment of cognitive and affective resources (Schunk, 1991; Chen et al., 2025). In addition, students with high academic self-efficacy are more likely to maintain adaptive academic attitudes when facing academic challenges and to employ more effective emotion regulation and self-management strategies, facilitating the translation of motivation into sustained engagement. Accordingly, we propose that under conditions of higher academic self-efficacy, the strength of the motivation–engagement association will increase—that is, academic motivation will correspond more strongly to higher levels of academic engagement.

Second, we propose that academic self-efficacy may moderate the association between university students' perceptual attitudes toward artificial intelligence (AI) and their academic engagement. Social cognitive theory indicates that individuals' behavior and motivation are shaped not only by internal personal factors (e.g., self-efficacy beliefs) but also by multiple environmental and situational influences, with technological tools constituting only one type of contextual cue (Bandura, 1997, 2001). As a key psychological resource, academic self-efficacy is significantly associated with students' task value appraisals, persistence, and levels of engagement, and it may shape how students cope with academic challenges (Wang and Zhang, 2024; Miao et al., 2025). Related evidence also indicates that individual differences in students' perceptual attitudes toward AI—such as perceived usefulness, ease of use, and impact salience—are associated with their academic-related behavioral performance (Wecks et al., 2024; Wu et al., 2025). Drawing on Technology Dependency Theory, we infer that when academic self-efficacy is high, students are more likely to maintain competence expectations and invest in self-regulatory effort; consequently, the association between stronger perceived AI attitudes and lower academic engagement may be attenuated. By contrast, when academic self-efficacy is low, stronger perceived AI attitudes may be more likely to align with reduced academic engagement. Indirect support for this proposition is provided by prior work showing that higher academic self-efficacy is typically linked to a stronger sense of meaning in academic and greater willingness to exert effort (Schunk and Zimmerman, 2008), and is associated with a weaker tendency toward dependence on technological tools (Zhang et al., 2024).

### 1.3 Research objectives

In summary, this study investigates the mediating role of academic motivation in the relationship between artificial intelligence (AI) perception and university students' academic engagement, as well as the moderating role of academic self-efficacy in both the direct and indirect relationships between AI perception and academic engagement. This study contributes to understanding the psychological mechanisms underlying the close relationship between AI perception and academic engagement under the moderation of academic self-efficacy.

## 2 Methods

### 2.1 Participants

A total of 1,484 college students were recruited from China. Convenience sampling was employed to invite students to participate in a questionnaire survey accessed via the platform Questionnaire Star (www.wjx.cn). The sample consisted of 823 females, accounting for 55.5% of the participants, and 661 males, representing 44.5% of the total. With regard to their geographical origin, 316 (21.3%) were from cities, a total of 1,168 (78.7%) originate from rural regions. With respect to their major fields, 68.6% are in the fields of science and engineering, while 31.2% are in the fields of humanities and social sciences.

### 2.2 Measures

#### 2.2.1 University students' AI-related perceptions

The University Students' AI-related Perceptions (USAP; Suh and Ahn, 2022) was utilized to assess college students' perceptions and attitudes toward artificial intelligence among college students. The scale includes 26 items in three dimensions: four items about cognitive (e.g., “School-based AI education is increasingly recognized as a critical component of twenty-first-century literacy”), 10 items about influence (e.g., “Empirical evidence consistently indicates that the benefits of artificial intelligence outweigh its potential drawbacks in educational settings”), 12 items about behavioral (e.g., “I possess a high level of artificial-intelligence self-efficacy”). Each item was rated on a 5-point Likert scale ranging from 1 (strongly disagree) to 5 (strongly agree). Item responses were coded so that higher scores consistently indicated more positive AI-related perceptions; therefore, any negatively worded items were reverse-coded prior to computing scale scores. Scale scores were calculated by averaging the relevant item responses to obtain subscale means and an overall mean score. The second-order CFA model showed that the ELS had good construct validity, with χ^2^/df = 6.774, RMSEA = 0.062, NFI = 0.962, GFI = 0.909, TLI = 0.959, CFI = 0.967. In this study, the overall scale's internal consistency reliability coefficient is 0.978, and the Cronbach's alpha coefficients for each dimension of cognitive, influence, behavioral were 0.931, 0.960, and 0.968, respectively.

#### 2.2.2 Academic motivation

Academic Motivation Scale (AMS; Biggs et al., 2001) was developed to assess college students' academic motivation. The scale consists of 20 items in two dimensions: deep academic motivation (10 items, e.g., “I am intrinsically motivated by discovering and understanding novel concepts during academic”), surface motivation (10 items, e.g., “I study to avoid failing the examination”). Each item was rated on a 5-point Likert scale ranging from 1 (completely inconsistent) to 5 (completely consistent). Item responses were coded such that higher scores consistently indicated higher levels of academic motivation (i.e., higher scores reflect stronger motivation), with higher subscale scores representing greater deep motivation or greater surface motivation, respectively. All items were keyed in the same direction; therefore, no reverse-coded items were involved. Scale scores were computed by averaging item responses to obtain an overall mean score as well as the mean scores for the deep- and surface-motivation subscales. The second-order CFA model showed that the ELS had good construct validity, with χ^2^/df = 5.458, RMSEA = 0.055, NFI = 0.975, GFI = 0.952, TLI = 0.971, CFI = 0.979. In this study, Cronbach's alpha was 0.934, and the Cronbach's alpha coefficients for deep motivation and surface motivation were 0.969 and 0.931, respectively.

#### 2.2.3 Academic self-efficacy

The academic self-efficacy was measured using the Chinese version of the Academic Self-Efficacy scale (AS; Pintrich and De Groot, 1990), revised by Liang and Zhou (2000) to fit the research context of Chinese students. The scale consists of 11 items (e.g., “I have found that I tend to daydream during class, which prevents me from focusing on the lecture”). Each item was rated on a 5-point Likert scale ranging from 1 (completely inconsistent) to 5 (completely consistent). Item responses were coded such that higher scores consistently indicated higher levels of academic self-efficacy. Because some items were negatively worded (e.g., daydreaming and difficulty concentrating), these items were reverse-coded prior to computing scale scores (i.e., 1 ↔ 5, 2 ↔ 4, and 3 remained unchanged). Scale scores were calculated by averaging all item responses after reverse-coding, with higher mean scores reflecting greater academic self-efficacy. The second-order CFA model showed that the ELS had good construct validity, with χ^2^/df = 4.002, RMSEA = 0.045, NFI = 0.992, GFI = 0.989, TLI = 0.985, CFI = 0.994. In this study, Cronbach's alpha coefficient for the scale was 0.777.

#### 2.2.4 Academic engagement

The Academic Engagement Scale (AES) was used to measure college students' academic engagement (Schaufeli et al., 2002). This scale contains 14 items and three subscales, namely, Vigor (five items, e.g., “When studying, I feel mentally energetic”), Dedication (four items, e.g., “I am enthusiastic about my studies”), and Absorption (five items, e.g., “Time flies when I am studying”). Each item was scored on a 7-point Likert scale [ranging from 0 (never) to 6 (always)]. Item responses were coded so that higher scores consistently indicated higher levels of academic engagement. All items were positively worded; therefore, no reverse-coded items were involved. Scale scores were computed by averaging item responses to obtain subscale mean scores (vigor, dedication, and absorption) and an overall mean score. The second-order CFA model showed that the ELS had good construct validity, with χ^2^/df = 7.959, RMSEA = 0.069, NFI = 0.984, GFI = 0.955, TLI = 0.979, CFI = 0.986. In this study, the Cronbach's alpha coefficient for the scale was 0.979, and the Cronbach's alpha coefficients for vigor, dedication, and absorption were 0.952, 0.966, and 0.955, respectively.

### 2.3 Procedure

This study employed a cross-sectional online survey design and was approved by the Research Ethics Committee of the authors' university. Written informed consent was obtained from all participants prior to data collection. In September 2025, participants completed an online questionnaire assessing academic engagement, academic self-efficacy, academic motivation, and AI-related perceptions via a widely used survey platform (Wenjuanxing; https://www.wjx.cn). Participants were informed that the survey was anonymous and confidential, and that they could withdraw at any time without penalty.

### 2.4 Data analysis

First, the present study computed descriptive statistics for the focal variables and conducted Pearson's correlation analyses among them (see Table 1). Second, Hayes (2013) PROCESS macro (Model 4) was employed to examine the statistical indirect effect of academic motivation in the association between AI-related perceptions and academic engagement (see Table 2). Given the cross-sectional design, this analysis was intended to characterize an indirect effect pattern at the statistical level rather than to make causal mediation inferences. Third, PROCESS (Model 15) was used to test whether academic self-efficacy moderated both the direct association and the conditional indirect association between AI-related perceptions and academic engagement (see Table 3). Significance of the direct and indirect effects was evaluated using a bootstrap procedure with 5,000 resamples.

**Table 1:** Correlational analyses for the study variable.

**Variables**	***M***	***D***	**1**	**2**	**3**	**4**
AI-related perceptions	2.09	0.617	1			
Academic engagement	4.52	1.138	−0.348^**^	1		
Academic motivation	3.13	0.643	−0.165^**^	0.441^**^	1	
Academic self-efficacy	3.26	0.651	−0.035	0.209^**^	0.485^**^	1

^*^p < 0.05.

^**^p < 0.01.

^***^p < 0.001.

**Table 2:** Testing the mediation effect of AI anxiety on academic motivation.

**Variables**	**Model 1 (academic motivation)**				**Model 2 (academic engagement)**				**Model 3 (academic engagement)**			
	β	**SE**	**LLCI**	**ULCI**	β	**SE**	**LLCI**	**ULCI**	β	**SE**	**LLCI**	**ULCI**
AI-related perceptions	−0.172	0.027	−0.225	−0.120	−0.521	0.042	−0.603	−0.44	−0.642	0.045	−0.73	−0.554
Academic motivation					0.698	0.039	0.619	0.776				
*R* ^2^	0.027				0.273				0.121			
*F*	41.731				277.315				204.183			

β are standardized coefficients.

SE, standard error; LLCI, lower limit of the 95% confidence interval; ULCI, upper limit of the 95% confidence interval.

^***^p < 0.001.

**Table 3:** Testing the moderated mediation effect of AI-related perceptions on academic engagement.

**Variables**	**Model 2 (academic engagement)**			
	β	**SE**	**LLCI**	**ULCI**
Academic motivation	0.720	0.047	0.629	0.812
Academic self-efficacy	−0.319	0.089	−0.495	−0.145
Academic motivation × academic self-efficacy	0.058	0.022	0.016	0.101
AI-related perceptions	−0.492	0.043	−0.576	−0.408
AI-related perceptions × academic self-efficacy	0.092	0.035	0.023	0.159
*R* ^2^	0.28			
*F*	115.028^***^			

β are standardized coefficients. Each column is a regression model that predicts the criterion at the top of the column.

SE, standard error; LLCI, lower limit of the 95% confidence interval; ULCI, upper limit of the 95% confidence interval.

^***^p < 0.001.

## 3 Results

### 3.1 Bivariate correlation analysis

Results of bivariate correlation analysis for the variables of interest were shown in Table 1. AI-related perceptions are significantly negatively correlated with academic engagement (*r* = −0.348, *p* < 0.01) and negatively with academic motivation (*r* = −0.65, *p* < 0.01). Academic motivation is significantly positively correlated with academic engagement (*r* = 0.441, *p* < 0.01) and with academic self-efficacy (*r* = 0.4855, *p* < 0.01). AI-related perceptions was not negatively correlated with academic self-efficacy (*r* = −0.035 *p* > 0.01). Therefore, hypothesis 1 was supported.

### 3.2 Mediating effect of academic motivation

We used Model 4 of the PROCESS macro (Hayes, 2013) to examine the mediating effect of academic motivation in the association between AI-related perceptions and academic engagement. As shown in Table 2, AI-related perceptions significantly and negatively predicted academic motivation (Model 1: β = −0.172, SE = 0.027, 95% CI [−0.225, −0.120], *p* < 0.001), and academic motivation significantly and positively predicted academic engagement (Model 2: β = 0.698, SE = 0.039, 95% CI [0.619, 0.776], *p* < 0.001). AI-related perceptions also showed a significant total effect on academic engagement (Model 3: β = −0.642, SE = 0.045, 95% CI [−0.730, −0.554], *p* < 0.001) and remained significant after controlling for academic motivation (direct effect; Model 2: β = −0.521, SE = 0.042, 95% CI [−0.603, −0.440], *p* < 0.001). By bootstrapping 5,000 samples, the indirect effect of AI-related perceptions on college students' academic engagement through academic motivation was significant (*ab* = −0.120, SE = 0.029, 95% CI [−0.179, −0.067]). Thus, academic motivation partially mediated the relationship between AI-related perceptions and academic engagement, accounting for 18.76% of the total effect.

### 3.3 Moderation effect of academic self-efficacy

**Figure 1:**
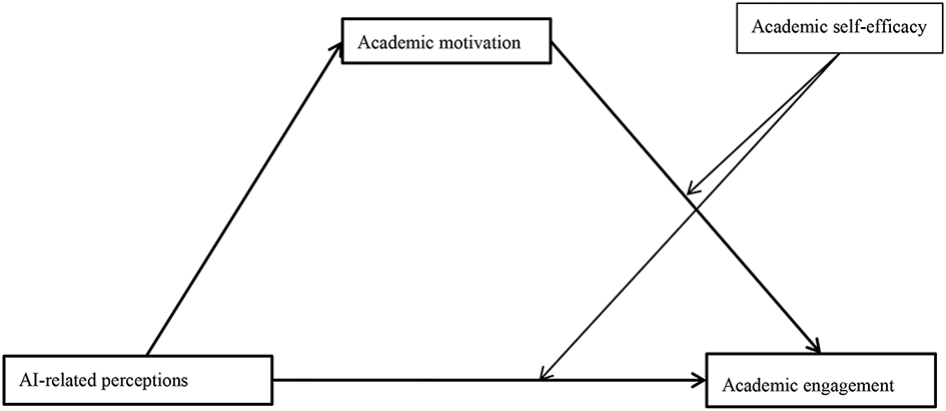
The assumed moderated mediation model.

**Figure 2:**
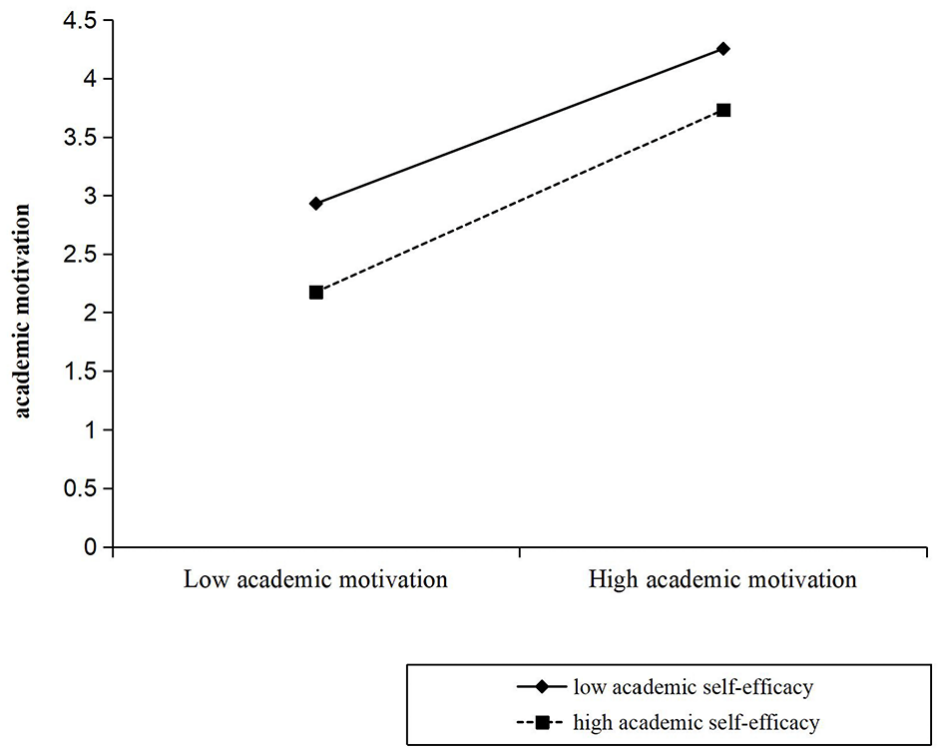
Academic self-efficacy moderates the indirect relationship between academic motivation and academic engagement.

We used Model 15 of the PROCESS macro (Hayes, 2013) to test the moderating effect of academic self-efficacy on the indirect and direct relationships between AI-related perceptions and academic engagement (see Figure 1). As shown in Table 3, academic motivation was positively correlated with academic engagement (β = 0.720*, p* < 0.001). The interaction between academic motivation and academic self-efficacy was also significant (β = 0.058, SE = 0.022, 95% CI [0.016, 0.101], *p* < 0.01; see Model 1). In other words, academic self-efficacy moderated the relationship between academic motivation and academic engagement. As shown in Figure 2, the relationship between academic motivation and academic engagement at low academic self-efficacy (M – 1 SD) and at high academic self-efficacy (M + 1 SD) was plotted. Simple slope tests showed that for students with high academic self-efficacy, the relationship between academic motivation and academic engagement was stronger (β_simple_ = 0.779, *p* < 0.001); while for students with low academic self-efficacy, the relationship between academic motivation and academic engagement was relatively weaker (β_simple_ = 0.662, *p* < 0.001).

**Figure 3:**
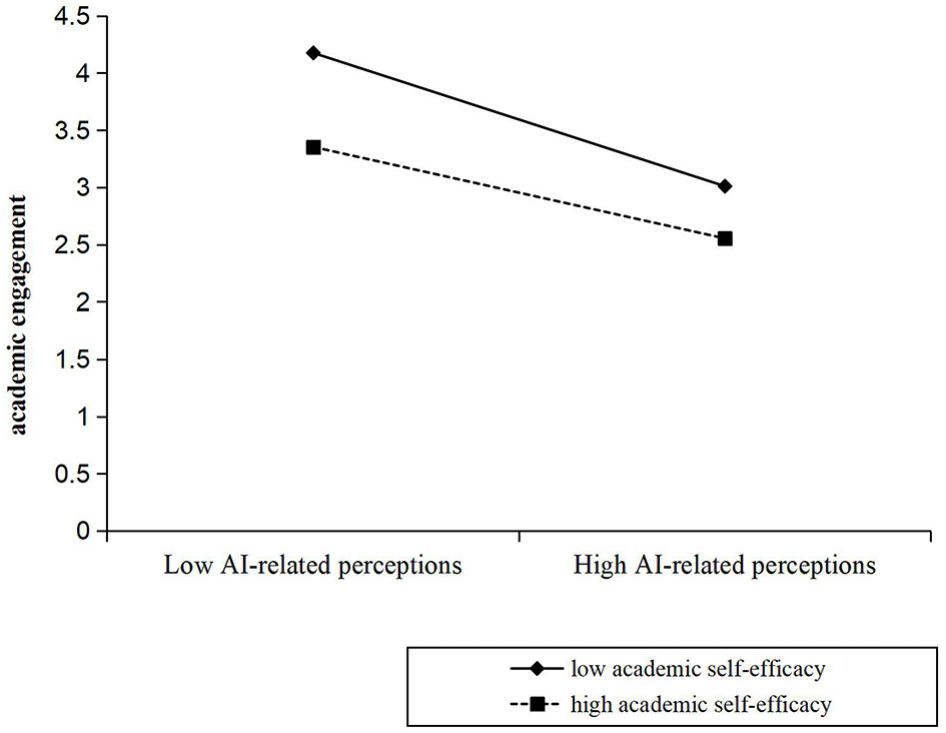
Academic self-efficacy moderates the indirect relationship between AI-related perceptions and academic engagement.

In addition, as shown in Table 3, AI-related perceptions was negatively correlated with academic engagement (β = −0.492, *p* < 0.001). The interaction between AI-related perceptions and academic self-efficacy was significant (β = 0.092, SE = 0.035, 95% CI [0.023, 0.159], *p* < 0.01; see Model 1). In other words, academic self-efficacy moderated the relationship between AI-related perceptions and academic engagement. As shown in Figure 3, the relationship between AI-related perceptions and academic engagement at low academic self-efficacy (M – 1 SD) and at high academic self-efficacy (M + 1 SD) was plotted. Simple slope tests showed that for students with low academic self-efficacy, the relationship between AI-related perceptions and academic engagement was stronger (β_simple_ = −0.583, *p* < 0.001); while for students with high academic self-efficacy, the relationship between AI-related perceptions and academic engagement was weaker (β_simple_ = −0.400, *p* < 0.001).

Finally, the bias-corrected percentile bootstrap analyses examined whether the indirect effect of AI-related perceptions on academic engagement via academic motivation was moderated by academic self-efficacy. Results showed that for students with low academic self-efficacy, the indirect relationship between AI-related perceptions and academic engagement was *weaker* (β = −0.088, SE = 0.039, 95% CI [−0.170, −0.016]), whereas for students with high academic self-efficacy, the indirect relationship between AI-related perceptions and academic engagement was *stronger (*β = −0.137, SE = 0.039, 95% CI [−0.222, −0.069]).

## 4 Discussion

Although empirical studies have reported an association between perceived artificial intelligence (AI) and university students' academic engagement (Tang et al., 2025a,b; Chan and Hu, 2023), relatively little is known about the psychological mechanisms underlying this relationship. Using a sample of university students, the present study examined (a) the mediating role of academic motivation in the association between perceived AI and academic engagement; and (b) the moderating role of academic self-efficacy in both the direct and indirect links. Pearson correlation analyses indicated that perceived AI was significantly and negatively associated with academic engagement. Mediation analyses further showed that academic motivation partially mediated the association between perceived AI and academic engagement. Moderation analyses revealed that academic self-efficacy significantly moderated both the direct path from perceived AI to academic engagement and the indirect path via academic motivation. Specifically, at lower levels of academic self-efficacy, the positive association between academic motivation and engagement was weaker, whereas the negative association between perceived AI and engagement was stronger. Conversely, at higher levels of academic self-efficacy, the positive motivation–engagement association was stronger, and the negative perceived AI–engagement association was weaker. These findings advance understanding of the perceived AI–engagement linkage and its plausible psychological pathways, highlighting the contingent role of academic self-efficacy.

### 4.1 Mediating role of academic motivation

The present study found a significant negative association between perceived attitudes toward artificial intelligence (AI) and academic motivation, whereas academic motivation was positively associated with university students' academic engagement. In other words, academic motivation exhibited a mediating effect in the relationship between perceived AI attitudes and academic engagement, thereby supporting our hypothesized mediation model. These results indicate that perceived AI attitudes are significantly related to students' academic engagement and are also indirectly linked to engagement via academic motivation. Collectively, the findings help clarify potential challenges associated with perceived AI in shaping students' engagement. In highly technology-supported academic environments, students' intrinsic academic motivation may be comparatively weaker, which may in turn be associated with reduced willingness to actively participate in academic activities and to engage in deep, effortful cognitive processing.

To further elucidate the mediating role of academic motivation in the link between perceived AI attitudes and university students' academic engagement, we discuss the two indirect pathways involved in the mediation model separately. First, the present study identified a significant negative association between perceived AI attitudes and academic motivation, indicating that students who report stronger perceptions of AI's usefulness, ease of use, and salience tend to exhibit relatively lower academic motivation. This pattern is consistent with prior findings (Bai and Wang, 2025; Chan and Hu, 2023). One plausible interpretation can be derived from the levels-of-processing framework (Craik and Lockhart, 1972), which highlights that academic outcomes depend on both time on task and the depth of cognitive processing. When university students perceive AI as a highly instrumental academic support tool, their academic may become more outcome-oriented and oriented toward rapid task completion, which can reduce opportunities for autonomous thinking and deep academic. Consequently, students may engage in shallower processing of academic materials, which may co-occur with diminished academic motivation (Craik and Lockhart, 1972; Zhai et al., 2024; Fan et al., 2025). In addition, Cognitive Load Theory indicatess that stronger perceptions of AI' s tool efficacy may, in certain task contexts, be associated with lower perceived cognitive load during academic (Jose et al., 2025; Gerlich, 2025). When cognitive demands are perceived as too low, students may experience insufficient challenge and reduced feelings of accomplishment, which can undermine interest and sustained effort and, in turn, relate to lower academic motivation.

Second, the present study found a significant positive association between academic motivation and university students' academic engagement. This pattern indicates that students with higher levels of academic motivation tend to exhibit greater engagement (i.e., they are more likely to invest more strongly in academic activities). Resource-regulation perspectives indicates that academic behavior is shaped by both external demands and the regulation of personal resources (DeShon et al., 1996; Seufert et al., 2024). Academic motivation represents a key internal driving force for academic and is likely linked to how individuals allocate limited resources such as attention, time, and effort (Shi, 2024; DeShon et al., 1996; Otermans et al., 2025). From this standpoint, highly motivated students may be more inclined to devote greater amounts of these resources to academic tasks, thereby predicting higher academic engagement. This finding is consistent with prior research (Li and Xue, 2023; Chen et al., 2023a,b). Moreover, academic motivation may be positively related to students' perceived task value, which can foster stronger attentional focus and greater enjoyment or immersion during the academic process, ultimately contributing to higher levels of academic engagement.

### 4.2 Moderating role of academic self-efficacy

Academic self-efficacy moderates the association between academic motivation and academic engagement. Specifically, the positive association between academic motivation and engagement is stronger among students with higher academic self-efficacy, whereas it is weaker among those with lower academic self-efficacy. These findings indicates that academic self-efficacy may amplify the beneficial role of academic motivation in predicting university students' academic engagement. According to self-efficacy theory, individuals with stronger efficacy beliefs are more likely to invest effort, persist in the face of difficulty, and sustain goal-directed behavior (Bandura, 1977). In this sense, for students with higher academic self-efficacy, motivation is more likely to translate into sustained engagement, supported by autonomous motivation and self-regulatory self-incentives, which are in turn positively associated with academic outcomes. Our results are consistent with prior evidence (Wang and Zhang, 2024; Meng and Zhang, 2023) showing that higher self-efficacy is typically linked to stronger academic motivation and, consequently, greater academic engagement. By contrast, students with lower academic self-efficacy may be more prone to academic-related anxiety and avoidance tendencies, which are associated with reduced engagement. Importantly, the present study also indicates that academic motivation is not the sole determinant of engagement and that the motivation–engagement linkage may be contingent on students' efficacy beliefs. As an internal psychological resource, academic self-efficacy shapes how students appraise their academic capability and is consequently related to the level of effort they invest in academic tasks.

Academic self-efficacy moderates the association between perceived artificial intelligence (AI) and academic engagement among university students. Specifically, among students with lower academic self-efficacy, stronger perceptions of AI are associated with lower levels of academic engagement. In contrast, students with higher academic self-efficacy tend to exhibit higher academic engagement when their perceived AI is weaker. Technology Dependency Theory indicates that a heightened propensity to rely on technological tools may be linked to shifts in individuals' motivation and behavioral regulation (Peters et al., 2018). Consistent with this account, prior evidence indicates that perceived AI is negatively related to students' academic behaviors (Nelson et al., 2025). When students perceive AI as highly useful, their intention to use it may increase; however, this may also be accompanied by weaker autonomous academic motivation and reduced engagement in deep processing, manifesting a tendency toward surface-level processing (i.e., shallow academic). In addition, academic self-efficacy—defined as individuals' beliefs about their capability to successfully perform academic tasks— may function as a moderator in students' academic processes (Moghadari-Koosha et al., 2020; Chen et al., 2025). Students with lower academic self-efficacy may lack sufficient confidence in their academic competence and, when confronted with the convenience afforded by AI, may be more likely to develop a stronger propensity for technology dependence. Such heightened reliance on AI tools is associated with reduced motivation to actively engage in academic tasks and may co-occur with lower levels of academic engagement.

## 5 Implication

This study identifies a significant negative association between students' AI perceptions and academic engagement and supports an indirect-effect pattern in which academic motivation mediates the link and academic self-efficacy moderates it. Given AI's growing ubiquity in higher education, restrictive or prohibitive approaches are neither feasible nor optimal. Accordingly, to mitigate technology-related risks while supporting deep learning, systematic educational reforms are warranted, and the following recommendations are proposed. First, at the level of institutional governance, higher education institutions should replace outcome-based prohibitions with a “transparent disclosure–reflective accountability” framework. Students should be required to document how AI was used (e.g., purpose and revision trajectory) when submitting AI-assisted work (Garcia Ramos, 2025; Weaver, 2024), while Institutions should require transparent AI-use disclosure and process evidence and grade primarily on critical analysis and verifiable student contribution, a design intended to curb cognitive offloading and protect deep engagement (Morrison and Richmond, 2020; Gerlich, 2025). In parallel, given individual differences in academic self-efficacy, institutions should implement early screening and tiered support for low-efficacy learners, using attribution retraining and strategy coaching to strengthen controllable attributions, competence expectations, and sustained engagement (Honicke and Broadbent, 2016; Försterling, 1985; Xu Z. et al., 2023). Second, at the level of instructional design, teachers should redesign assignments and assessments that are easily substitutable by AI, shifting toward cognitively demanding tasks that are authentic, open-ended, and iterative (Donker et al., 2014; Theobald, 2021; Wiggins, 1990; Bower et al., 2024), such that students must still engage in problem framing, evidence evaluation, and reflective revision even when leveraging AI. Feedback should be motivationally informed by Self-Determination Theory, systematically supporting autonomy, competence, and relatedness, and emphasizing process-focused recognition to reinforce perseverance and metacognitive regulation. Third, at the level of learner development, metacognitive monitoring of AI use should be integrated into core academic competencies by training students to apply the self-regulated academic cycle (planning–monitoring–evaluating–adjusting) to AI-supported study (Chiu et al., 2024; Evangelista, 2025; Khlaif et al., 2025; Liu et al., 2025; Panadero, 2017; Xia et al., 2026), including goal calibration and source credibility appraisal. Drawing on Social Cognitive Theory, institutions should also cultivate peer modeling communities in which effective users share growth-oriented narratives and transferable strategies, providing vicarious experiences and practicable scripts that strengthen self-efficacy and more adaptive beliefs about the technology–academic relationship.

## 6 Limitations and future directions

The present study has several limitations that warrant further investigation. First, the cross-sectional design precludes establishing temporal ordering and causal directionality; accordingly, the findings should be interpreted as statistical associations and an indirect-effect pattern rather than evidence of causal mediation. Future research should employ longitudinal designs to test the temporal precedence and robustness of the proposed mechanism. Second, the sample was restricted to Chinese undergraduates, and the specific cultural and educational context may constrain external validity; replication across regions, institution types, and cross-cultural samples is needed. Third, our assessment of AI focused on learners' perceptual attitudes toward AI rather than directly capturing actual use behaviors (e.g., frequency, usage patterns, or dependence), which limits behavioral inferences; subsequent studies should incorporate objective usage indicators (e.g., platform logs) and more contextualized, behavior-sensitive measures. Finally, we did not control for potential confounds such as gender, disciplinary background, or year of study, which may bias effect estimates and boundary conditions; future work should include theoretically justified covariates and/or conduct multi-group and interaction tests to strengthen internal validity and explanatory precision. Moreover, the academic self-efficacy scale exhibited relatively low internal consistency in the current sample (e.g., Cronbach' s α), measurement error may be inflated, which could yield unstable interaction-term estimates and potentially attenuate (i.e., underestimate) the moderation effect or introduce bias. Future research should adopt self-efficacy measures with stronger reliability and clearer factor structures, and test measurement invariance based on a more explicitly specified measurement model. In addition, measurement error could be modeled explicitly using structural equation modeling (SEM) or latent interaction frameworks to enhance the robustness and interpretability of the moderation findings.

Despite providing useful insights into the relations among perceived AI, academic motivation, academic self-efficacy, and academic engagement, several directions remain open. First, longitudinal research should examine whether the association between perceived AI and engagement varies across academic years, and whether such patterns differ by levels of academic self-efficacy, thereby clarifying how self-efficacy conditions the perceived AI–engagement linkage and informing targeted educational interventions. Second, future studies should further unpack the interactive mechanisms between perceived AI and academic self-efficacy—for instance, by testing whether additional psychological variables (e.g., metacognitive monitoring, self-regulated academic, achievement goals, or autonomy need satisfaction) operate as mediators in the perceived AI–engagement process. Third, given the rapid expansion of AI applications, it is important to extend investigation across disciplinary domains and to test whether disciplinary characteristics moderate students' perceptions and their engagement outcomes, as fields may differ systematically in academic practices, motivational profiles, and vulnerability to technology reliance.

## Data Availability

The original contributions presented in the study are included in the article/supplementary material, further inquiries can be directed to the corresponding authors.
